# Enhanced Tumor‐Targeted Delivery of Arginine‐Rich Peptides via a Positive Feedback Loop Orchestrated by Piezo1/integrin β1 Signaling Axis

**DOI:** 10.1002/advs.202409081

**Published:** 2024-09-11

**Authors:** Minghai Ma, Xing Li, Minxuan Jing, Pu Zhang, Mengzhao Zhang, Lu Wang, Xiao Liang, Yunzhong Jiang, Jianpeng Li, Jiale He, Xinyang Wang, Min Lin, Lei Wang, Jinhai Fan

**Affiliations:** ^1^ Key Laboratory of Environment and Genes Related to Diseases, Ministry of Education, Department of Urology, The First Affiliated Hospital Xi'an Jiaotong University Xi'an 710061 China; ^2^ Department of Thoracic Surgery, Tangdu Hospital Air Force Medical University Xi'an 710038 China; ^3^ Key Laboratory of Biomedical Information Engineering, Ministry of Education, Bioinspired Engineering and Biomechanics Center (BEBC), School of Life Science and Technology Xi'an Jiaotong University Xi'an 710049 China

**Keywords:** arginine‐rich peptides, integrin β1, piezo1, positive feedback loop, tumor‐targeted delivery

## Abstract

Peptide‐based drugs hold great potential for cancer treatment, and their effectiveness is driven by mechanisms on how peptides target cancer cells and escape from potential lysosomal entrapment post‐endocytosis. Yet, the mechanisms remain elusive, which hinder the design of peptide‐based drugs. Here hendeca‐arginine peptides (R11) are synthesized for targeted delivery in bladder carcinoma (BC), investigated the targeting efficiency and elucidated the mechanism of peptide‐based delivery, with the aim of refining the design and efficacy of peptide‐based therapeutics. It is demonstrated that the over‐activated Piezo1/integrin β1 (ITGB1) signaling axis significantly facilitates tumor‐targeted delivery of R11 peptides via macropinocytosis. Furthermore, R11 peptides formed hydrogen bonds with integrin β1, facilitating targeting and penetration into tumor cells. Additionally, R11 peptides protected integrin β1 from lysosome degradation, promoting its recycling from cytoplasm to membrane. Moreover, this findings establish a positive feedback loop wherein R11 peptides activate Piezo1 by increasing membrane fusion, promoting Ca^2+^ releasing and resulting in enhanced integrin β1‐mediated endocytosis in both orthotopic models and clinical tissues, demonstrating effective tumor‐targeted delivery. Eventually, the Piezo1/integrin β1 signaling axis promoted cellular uptake and transport of peptides, establishing a positive feedback loop, promoting mechanical delivery to cancer and offering possibilities for drug modification in cancer therapy.

## Graphical abstract

Arginine‐rich peptides facilitate targeted‐delivery by forming hydrogen bonds with integrin β1 and promoting its recycling from cytoplasm to membrane. The Piezo1/integrin β1 signaling axis enhances cellular uptake and transport of peptides through increasing membrane fusion and Ca^2+^ releasing, which ultimately establishes a positive feedback loop, promoting mechanical delivery to cancer and offering possibilities for drug modification in cancer therapy.

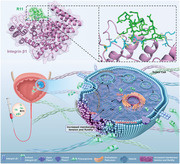

## 1 Introduction

In the past decades, the field of drug delivery has witnessed significant progress through the adoption of peptide‐based vehicles for the delivery of therapeutic agents to tumor sites.^[^ ^1^, ^2^ ^]^ Despite these advancements, challenges persist in achieving optimal effectiveness, such as retention in blood circulation, navigating dense barriers in the extracellular matrix, avoiding off‐target effects and preventing lysosomal entrapment during endocytosis.^[^ ^3^ ^]^ Researchers continually explore novel ways to improve tumor delivery through developing new peptides.^[^ ^4^, ^5^ ^]^ Despite these efforts, a considerable gap still exists in revolutionizing peptide‐based drug delivery for cancer therapy.

While peptides like TAT facilitate the transfer of biological macromolecules into cells, they lack the capacity to target specific receptors in cells, like integrin family.^[^ ^6^, ^7^ ^]^ Integrins, crucial for cell‐matrix interactions,^[^ ^8^, ^9^, ^10^ ^]^ have been targeted in cancer therapy, with the RGD peptide being a notable example of targeted integrin binding.^[^ ^11^, ^12^ ^]^ However, challenges such as potential conformational shifts of integrins, undesirable physicochemical properties, and adverse reactions due to zwitterionic or amphoteric design hinder the success of the design of RGD‐based drugs targeting integrin.^[^ ^11^, ^13^ ^]^ These limitations necessitate the exploration of alternative integrin‐targeting strategies. We previously synthesized hendeca‐arginine peptides (R11) conjugates carrying various payloads for tumor‐targeted delivery in bladder carcinoma (BC),^[^ ^14^, ^15^, ^16^ ^]^ which composed of 11 arginines and exhibited enhanced uptake efficiency and lysosomal escape in cancer. While promising, the lack of clarity regarding how R11 peptides interact with integrin and subsequent endocytosis mechanisms further impedes the development of R11‐based drugs with enhanced cellular uptake.

Efficient cellular uptake also requires endocytosis vehicles to transport peptides. This enhancement has been reported to be associated with Ca^2+^ signal that induces vesicle fusion during endocytosis by manipulating membrane curvature.^[^ ^17^, ^18^ ^]^ Arginine‐rich peptides can induce plasma membrane deformation, such as protrusions, bifurcations, and multilamellarity.^[^ ^19^, ^20^ ^]^ This membrane deformation may initiate Ca^2+^ signal, originating from Piezo1, a mechanically activated ion channel.^[^ ^21^, ^22^ ^]^ Moreover, integrin β1 (ITGB1) can up‐regulate piezo1 function, contributing to cellular endocytosis.^[^ ^23^, ^24^ ^]^ However, the specific correlation between peptides and the vesicle fusion process modulated by Piezo1/ITGB1 needs systematic investigation.

Our work suggested the involvement of the Piezo1/ITGB1 signaling axis in the tumor‐targeted delivery of R11 peptides. However, the specific targeting and transport process remain unclear. This study aims to synthesize R11 peptides and explore their targeting ability and uptake mechanism mediated by the Piezo1/ITGB1 signal axis. Additionally, the mechanism and process of integrin recycling will be studied. Understanding the biological process and mechanisms of peptides delivery will provide novel insights into addressing the integrin‐targeting challenges and contribute to the development of effective cancer therapies.

## 2 Results

### 2.1 Arginine‐Rich Peptides Facilitate Targeted‐Delivery in Cancer

**Figure 1:**
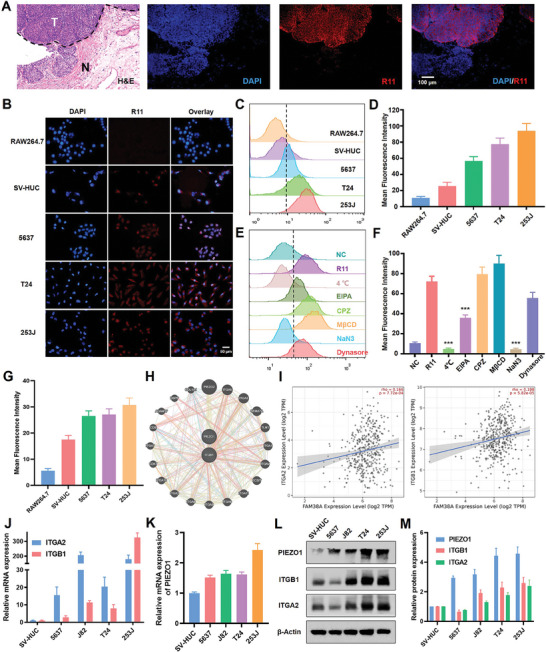
Arginine‐rich peptides facilitate targeted‐delivery in cancer. A) Fluorescence in situ hybridization (FISH) of clinical tissue from a bladder cancer patient stained with R11 peptides. B) Fluorescence assay showed different cells treated with R11 peptides (BC cell lines: 5637, T24, and 253J; Macrophage cell line: Raw264.7; Normal urothelial cell line: SVHUC‐1). C) Flow cytometry results of different cells treated with R11 peptides (100 nM). D) Quantitative data from Flow cytometry results of different cells treated with R11 peptides. E) Flow cytometry results of T24 cells treated with different endocytosis inhibitors (EIPA: Macropinocytosis; CPZ: Clathrin‐mediated endocytosis, MβCD: Caveolin‐mediated endocytosis; NaN3: Energy inhibitor; Dynasore: Dynamin inhibitor). F) Quantitative data of cells treated with different endocytosis inhibitors. G) Quantitative data of cells treated with R11 peptides in fluorescence experiment. H) Interaction network between ITGB1 and Piezo1(FAM38A) from GeneMANIA database. I) Correlation between ITGA2, ITGB1 and Piezo1 from TIMER database. J) Relative RNA expression of ITGA2, ITGB1 and PIEZO1 K) in SVHUC‐1, 5637, J82, T24, 253J cells. L) Expression of ITGA2, ITGB1 and PIEZO1 in protein level in different cells. M) Quantitative data of relative protein expression in cells.

To explore the targeting behavior, we synthesized hendeca‐arginine peptide (R11) using the solid‐phase peptide synthesis method. We then asked whether the R11 peptides retained the specific tumor‐targeted ability. Utilizing fluorescence in situ hybridization (FISH) on clinical tissue from a patient with T1‐stage bladder cancer (BC), we demonstrated that R11 peptides accurately identified the BC margin and distinguished between tumor tissue and normal mucosa (**Figure** **1**A). To further evaluate the targeting ability, three BC cell lines (5637, T24, and 253J) were incubated with R11, while a macrophage cell line (Raw264.7) and a normal urothelial cell line (SV‐HUC) served as a negative control. Fluorescence microscopy (Figure 1B) and flow cytometry (Figure 1C) demonstrated a stronger fluorescence signal in BC cell lines compared to control cells. The flow cytometry quantitatively conformed these observations (Figure 1D).

To elucidate the peptide uptake pathway, several endocytosis inhibitors were used including EIPA, chlorpromazine (CPZ), Methyl‐β‐cyclodextrin (MβCD), Dynasore, NaN_3_. The uptake of R11 proved to be an energy‐dependent process, blocked by NaN_3_ and 4 °C. This uptake process was suppressed nearly half by EIPA, an inhibitor of macropinocytosis (Figure 1E). The quantitative result of flow cytometry was shown in Figure 1F. Dynasore, an inhibitor of GTPase dynamin involved in clathrin and caveolin mediated endocytosis also inhibited uptake partly. Therefore, macropinocytosis was identified as the primary endocytosis pathway for arginine‐rich peptides. And the quantitative result of fluorescence in Figure 1B was shown in Figure 1G.

To assess the potential mechanism of the uptake, RNA sequencing (RNA‐Seq) was employed to evaluate the key protein or receptors in our previous study. The ITGA2, ITGB1 and Piezo1 genes, associated with mechanical transport, exhibited downregulation following treatment with R11 peptide modified gold nanoparticles according to our previous study (Figure S1, Supporting Information). This regulation might play a mediating role in peptide‐nanoparticle delivery. Additionally, ITGB1 and Piezo1 showed a significant interaction, collectively influencing the function of Collagen (COL1A1), CD9, immunology and fibroblasts microenvironment (Figure 1H). The result obtained from the TIMER database indicated a linear correlation between the expression of ITGA2 and ITGB1 with Piezo1(FAM38A), suggesting their potential collective involvement in peptides uptake (Figure 1I). Subsequent molecular biology experiments were conducted to explore the expression and function of the Piezo1/integrin β1 axis in the delivery of peptides. ITGA2, ITGB1 and Piezo1 in BC cells was confirmed by both qRT‐RCR (Figure 1J,K) and western blotting results (Figure 1L,M), which were overexpressed in BC cells and mediated the sense mechanical stimuli of membrane. In summary, piezo1/integrin β1 axis was implicated in peptide delivery, which was over‐activated in BC and might hold potential as a therapeutic approach for tumor‐targeted delivery.

### 2.2 Piezo1/Integrin β1 Axis Enhances the Targeted Delivery

**Figure 2:**
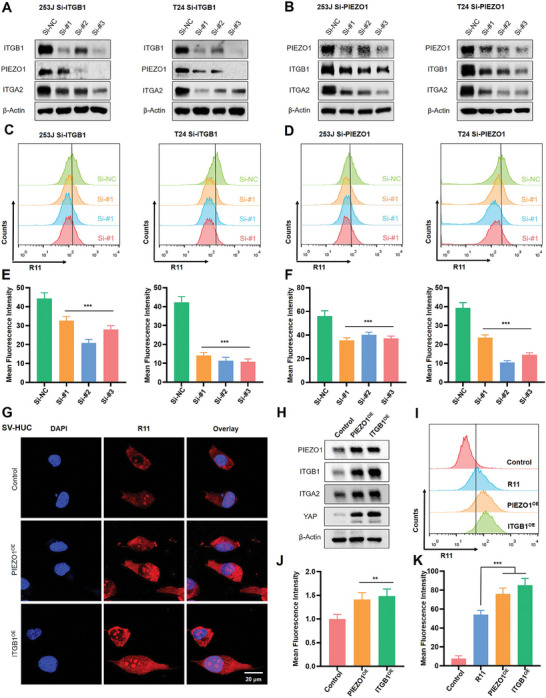
Piezo1/integrin β1 axis synergistically enhances the targeted delivery. A) Expression of ITGB1, PIEZO1, ITGA2 after knocking down ITGB1 in 253J and T24 cell lines. B) Expression of ITGB1, PIEZO1, ITGA2 after knocking down Piezo1 in 253J and T24 cell lines. C) Uptake efficiency of R11 peptides from flow cytometry after knocking down ITGB1 in 253J and T24 cell lines. D) Uptake efficiency of R11 peptides from flow cytometry after knocking down PIEZO1 in 253J and T24 cell lines. E) Quantitative data of flow cytometry after treatment with R11 peptides under knocking down ITGB1 cells. F) Quantitative data of flow cytometry after treatment with R11 peptides under knocking down PIEZO1 in cells. G) Fluorescence assay of SV‐HUC cells overexpressed PIEZO1 and ITGB1 treated with R11 peptides. H) Western blotting result of SV‐HUC cells overexpressed PIEZO1 and ITGB1. I) The uptake efficiency of peptides from flow cytometry in PIEZO1^OE^ and ITGB1^OE^ cells. J) The quantitative data of fluorescence images in Figure 2G. K) The quantitative data of flow cytometry in Figure 2I.

To further investigate the influence of Piezo1/integrin β1 on R11 uptake, we knocked down the expression of Piezo1 and ITGB1 gene in T24 and 253J cells using small interfering RNA (si‐RNA). As depicted in **Figure** **2**A and B, the suppression of ITGB1 was observed when Piezo1 was knocked down, and vice versa, indicating mutual modulation of Piezo1/integrin β1 expression. The quantitative data was shown in Figure S2 (Supporting Information). Subsequent flow cytometry analysis revealed significant inhibition of R11 peptide uptake when the Piezo1/integrin β1 axis was knocked down in T24 and 253J cells (Figure 2C,D), with quantitative results represented in Figure 2E,F. Then the normal cell lines SV‐HUC was engineered to overexpress piezo1 and ITGB1 through the lentiviral transfection. As depicted in Figure 2G, the results illustrated a notable enhancement in the fluorescence signal of peptides in PIEZO1^OE^ and ITGB1^OE^ cells (PIEZO1^OE^ for Piezo1 overexpression and ITGB1^OE^ for ITGB1 overexpression). Western blotting result verified the successful overexpression of PIEZO1 and ITGB1 (Figure 2H; Figure S3, Supporting Information), further indicating the synergistic effect of Piezo1/integrin β1 axis. The result from flow cytometry also supported the enhanced uptake of peptides in both PIEZO1^OE^ and ITGB1^OE^ cells (Figure 2I). The quantitative results of fluorescence and flow cytometry were detailed in Figure 2J,K. Collectively, ITGB1 and PIEZO1 have been observed to display a coordinated expression profile in various cell lines, irrespective of being overexpressed or suppressed. This consistent behavior suggested a tightly regulated mechanism governing the levels of related proteins, which could play a significant role in the delivery of peptides, offering new avenues for cancer treatment.

### 2.3 Integrin β1 Provides Binding Sites and Activates Downstream Signal

**Figure 3:**
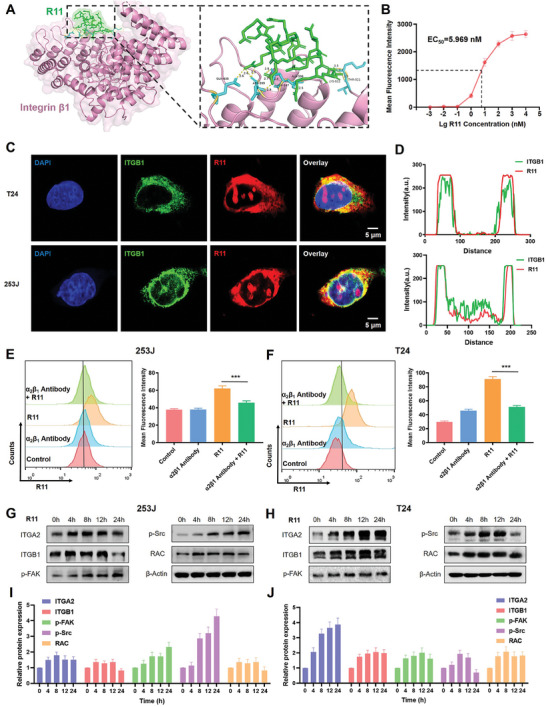
Integrin β1 provides binding sites for peptides and activates downstream signal. A) Molecular docking scheme illustrating the interaction between integrin β1 and R11 peptides. B) The binding affinity between β1 and peptides from ELISA assay. C) Fluorescence assay depicting the co‐localization between integrin β1 and R11 peptides in T24 and 253J cells (Red: R11; Green: Integrin β1; Blue: DAPI). D) Quantitative curve of the fluorescence assay. E) Flow cytometry and quantitative data of 253J cells blocked by integrin α2β1 and treated with R11 peptides. F) Flow cytometry and quantitative data of T24 cells blocked by integrin α2β1 and treated with R11 peptides. G) Western blotting results of ITGA2/ITGB1/FAK/Src/RAC proteins in 253J cells treated with R11 peptides. H) Western blotting results of various proteins in T24 cells treated with R11 peptides. I,J) The quantitative data of western blotting assay in cells treated with peptides.

To investigate how R11 peptides interact with the Piezo1/integrin β1 axis, we first predicted and calculated their binding ability separately using molecular docking (**Figure** **3**A). Results indicated that R11 peptides strongly interacted with integrin β1 through multiple hydrogen bonds at GLN‐635, ASN‐599, ASP‐597 and others, with a low binding energy (−204.7 kcal mol^−1^). However, Piezo1 did not exhibit binding affinity with R11 peptides, which might influence the uptake of peptides indirectly. Then ELISA assay was conducted to determine the binding affinity between peptides and integrin β1, exhibiting a strong affinity with an EC_50_ value of 5.969 nM as depicted in Figure 3B. Next, immunofluorescence assay demonstrated co‐localization of integrin β1 (Green) and R11 (red) in T24 and 253J cell lines (Figure 3C), as confirmed by the quantitatively analysis (Figure 3D). Then the integrin α2β1 antibody was employed to block the binding motif of integrin α2β1. The results from the western blotting showed a decrease in the levels of integrin β1 and Piezo1, p‐FAK, p‐Src, YAP, and RAC, while there was no observed decrease in integrin αVβ3, indicating the specificity in blocking integrin β1 (Figure S4A, B, Supporting Information). Then the fluorescence assay exhibited that the uptake ability of peptides was inhibited when integrin α2β1 was blocked, as visualized by immunofluorescence stain in (Figure S4C, D, Supporting Information). Along with the qualitative assessment through imaging, we have performed quantitative analysis to measure the degree of R11 peptide uptake in the presence of the blocking antibody (Figure S4E, F, Supporting Information). Flow cytometry also revealed that the uptake of R11 peptides was completely inhibited, which further verified the interaction ability of integrin β1 with R11 peptides in 253J and T24 cell lines (Figure 3E,F). Additionally, western blotting identified activation of integrin α2β1 and downstream signaling pathways FAK/SRC/RAC, further enhancing cellular adhesion, motility and endocytosis (Figure 3G,H). The quantitative data was shown in Figure 3I,J. Similarly, the RT‐qPCR results also verified that peptides activated the expression of ITGA2, ITGB1, and PIEZO1 (Figure S5, Supporting Information). These findings imply that the formation of the R11‐integrin β1 complex may occur after R11 recognizes the integrin β1 on the membrane surface, subsequently activating downstream FAK/SRC/RAC signal to modulate cell behaviors and facilitating cellular uptake of peptides.

### 2.4 Integrin β1 Mediates Intracellular Recycling and Avoids Degradation by Lysosomes

**Figure 4:**
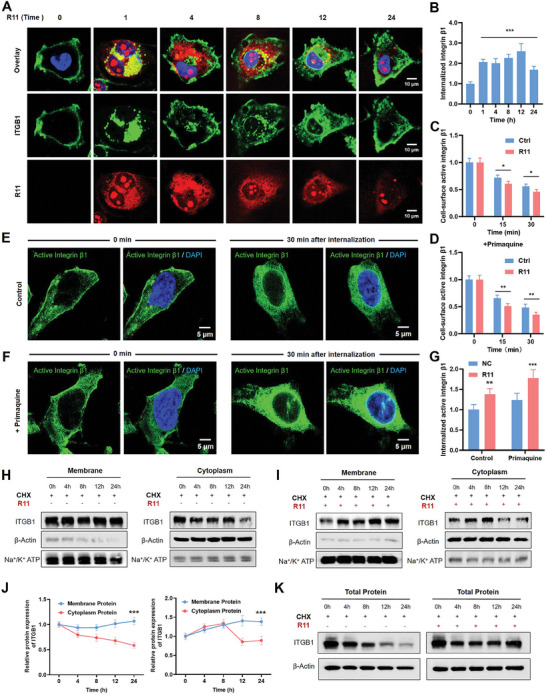
Integrin β1 mediates intracellular recycling and prevents lysosomes degradation. A) Fluorescence assay of ITGB1 and R11 peptides in T24 cells treated with R11 for 0, 4, 8, 12, 24 h. B) The quantitative analysis of internalized integrin β1 in fluorescence image. C) The cell‐surface levels of active integrin β1 from flow cytometry following the initiation of endocytosis at 15 and 30 minutes. D) The cell‐surface levels of active integrin β1 from flow cytometry following the initiation of endocytosis with the presence of primaquine (100 µM). E, F) Fluorescence image of active integrin β1 in cells treated with peptides and primaquine for 30 min. G) The quantitative result of internalized active integrin β1 from fluorescence images. H, I) Western blotting result of membrane and cytoplasmic proteins of integrin β1 in cells treated with CHX and R11 peptides. J) The quantitative result of western blotting in membrane and cytoplasm protein isolation experiment. K) Western blotting result of ITGB1 protein in cells treated with cycloheximide (CHX) and R11 peptides.

Typically, peptide‐based nanoparticles may undergo endocytosis, eventually entering late endosomes or lysosomes, where degradation by various enzymes and acidic pH occurs — a critical step in drug delivery.^[^ ^25^, ^26^ ^]^ Our previous studies found that arginine‐rich peptides can facilitate the escape of nanoparticles from lysosomes to cytoplasm, protecting nanoprobes from degradation. However, the specific mechanism remains unclear. Here we used fluorescence assay and flow cytometry to observe the uptake process of integrin β1. As depicted in **Figure** **4**A, integrin β1 (Green) initially anchored on the membrane and gradually internalized in concert with R11 (Red). Over time, the integrin β1 signal was observed to diffuse progressively within the cytoplasm, culminating in its recycling back to the cell membrane, while the R11 signal exhibited a gradual decline. Notably, R11 demonstrated stable targeting of the nucleolus throughout the process, presenting a unique advantage for gene‐targeted therapy. The quantitative data revealed that the internalized integrin β1 increased with time and decreased at last, indicative of a dynamic recycling mechanism (Figure 4B).

To delve deeper into the recycling role of integrin β1, the recycling inhibitor primaquine was employed in both flow cytometry and fluorescence assays.^[^ ^27^, ^28^ ^]^ The flow cytometry data revealed a notable decrease in the cell‐surface levels of active integrin β1 following the initiation of endocytosis at 15 and 30 minutes, with R11 peptides present (Figure 4C; Figure S6A, Supporting Information). The presence of primaquine accentuated the reduction in active integrin β1 on the cell surface, indicating that R11 peptides facilitated the recycling of integrin β1 (Figure 4D; Figure S6B, Supporting Information). However, there was no obvious decrease in inactive integrin β1 after endocytosis was triggered (Figure S6C, D, Supporting Information). Furthermore, the internalization of active integrin β1 was significantly enhanced by R11 peptides (Figure 4E). Inhibition of receptor recycling with primaquine magnified the difference in active integrin β1 internalization in cells treated with peptides (Figure 4F). The quantitative result was displayed in Figure 4G, further validating the role of peptides in the cellular recycling of active integrin β1.

To further determine the cellular distribution of integrin β1, we performed membrane and cytosol protein isolation experiment. Cycloheximide (CHX) was employed to inhibit protein synthesis, thereby eliminating the confounding effects of endogenous protein production on the experimental outcomes. As depicted in Figure 4H, the expression level of integrin β1 on the membrane remained nearly constant, while cytoplasmic protein degraded gradually under CHX treatment. This indicated that integrin β1 naturally recycled from cytoplasm to membrane. Notably, when R11 peptides persisted, integrin β1 on the membrane increased significantly, initially rising in the cytoplasm and then decreasing (Figure 4I). And the quantitative analysis was presented in Figure 4J. These results collectively suggest that R11 peptides stimulate integrin β1 recycle from cytoplasm to membrane, thereby positively enhancing the targeted delivery mediated by integrin β1.

As illustrated in Figure 4K, R11 peptides extend the degradation cycle and increase the stability of integrin β1 compared to CHX group, with the quantitative data presented in Figure S6E (Supporting Information). To explore deeper into the degradation pathway, proteasome inhibitor MG132 and lysosomal inhibitor Chloroquine (CQ) were used to treat cells for 24 h in the degradation experiment. Results revealed that R11 could notably protect integrin β1 from degradation when CQ inhibited lysosome function, suggesting that peptides uptake might intervene the lysosomal pathway (Figure S6F, G, Supporting Information). Taken together, R11 peptides protected integrin β1 from lysosome degradation, promoting its recycling from cytoplasm to membrane and establishing a positive feedback loop in tumor‐targeted delivery.

### 2.5 R11 Peptide Activates Piezo1/YAP and Facilitates Endocytosis

**Figure 5:**
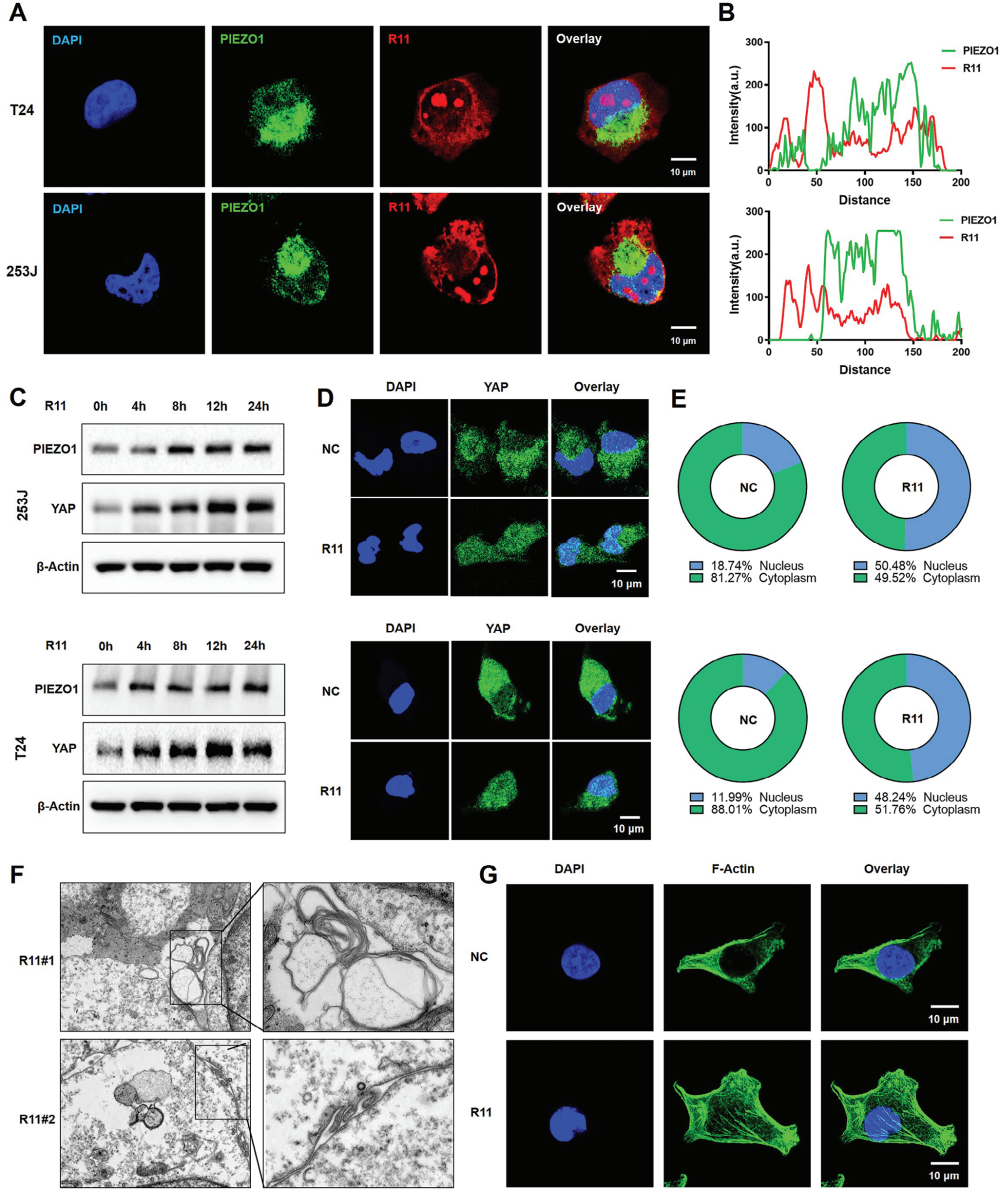
R11 peptide activates Piezo1/YAP and facilitates endocytosis. A) Fluorescence assay of the distribution of Piezo1 and R11 peptides in 253J and T24 cells. (Red: R11; Green: Piezo1; Blue: DAPI). B) Quantitative curve of the fluorescence assay in 253J and T24 cells. C) Western blotting results showing Piezo1/YAP expression in 253J and T24 cells treated with R11 peptides. D) Immunofluorescence of YAP signal in the cytoplasm and nucleus after R11 treatment. (Green: YAP; Blue: DAPI). E) Quantitative data of nucleo‐cytoplasmic ratio of YAP. F) Transmission electron microscope (TEM) image of cells treated with R11 peptides. G) Fluorescence assay of F‐Actin in cells treated with R11 peptides.

In addition to integrin β1, Piezo1 also plays an important role in peptides uptake. As mentioned earlier, prediction from AutoDock software indicated that R11 peptides could not directly interact with the mechanical ion channel Piezo1. This observation was further supported by an immunofluorescence assay, revealing no significant co‐localization between Piezo1 and R11 peptides in T24 and 253J cells (**Figure** **5**A,B). Western blotting results demonstrated that R11 peptides activated the expression level of both Piezo1 and YAP (Figure 5C). The quantitative data was shown in Figure S7A (Supporting Information). YAP, function as the downstream signal of Piezo1, was activated by R11 peptides entering into the nucleus (Figure 5D,E), collectively enhancing the mechanical sensing and uptake of tumor cells.

The Piezo1/YAP as the mechanical ion channel is capable of sensing mechanical tension and morphological alterations in the membrane and thus facilitate endocytosis. Therefore, transmission electron microscope (TEM) was used to observe the membrane structure following treatment with R11 peptides. R11 induced membrane to form a multilayer structure and sprouting protuberance, indicative of endocytosis (Figure 5F). Additionally, staining for phalloidin (F‐Actin), representing stress fiber architecture, indicated that R11 peptides facilitated cell mechanical properties (Figure 5G), promoting favorable conditions for tumor cells to uptake. R11 peptides were also observed to activate the expression of α‐Smooth Muscle Actin (α‐SMA), a downstream signal for Piezo1 and a key marker of contractile function in cells (Figure S7B, Supporting Information). Previous reports have indicated that arginine‐rich peptides like R9 and R12 stimulate dynamic morphological alterations and increase membrane curvature, ultimately enhancing cellular uptake efficiency.^[^ ^19^, ^20^ ^]^ The insertion of arginine peptides into the lipid bilayer can disrupt its structural integrity, causing local curvature and deformation that may lead to the formation of membrane protrusions or invaginations. In this study, we reported that R11 peptides induced the formation of a multilayered membrane and protuberances, possibly facilitating the creation and transit of endocytic vesicles, which could enhance the endocytic process and, by extension, the delivery of therapeutic agents to tumor cells.

### 2.6 Piezo1/Integrin β1 Collectively Promotes Peptides Delivery via Activation of Ca^2+^ and YAP Signal

**Figure 6:**
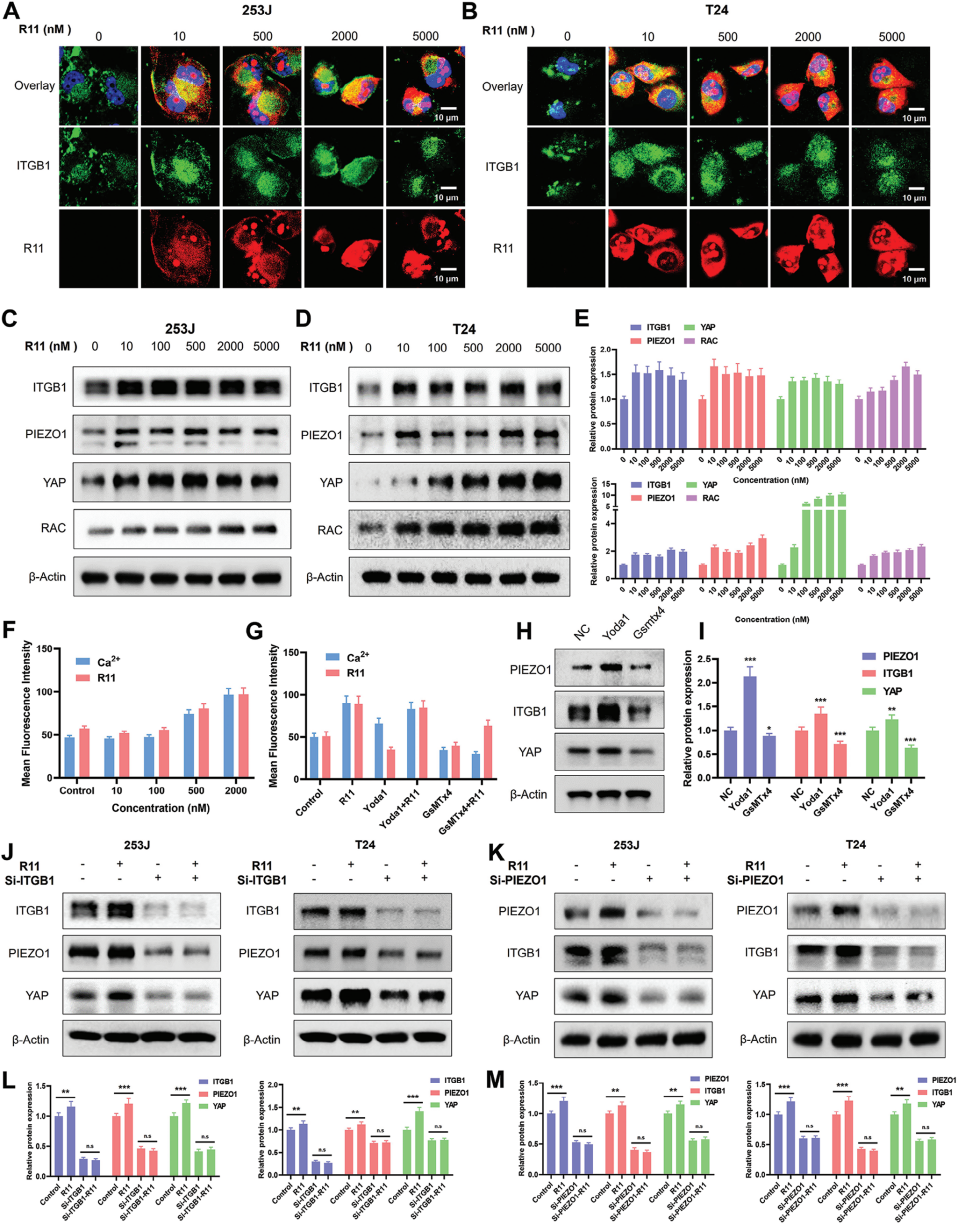
Piezo1/integrin β1collectively activates Ca^2+^ and YAP signal to promote peptides delivery. A) Fluorescence assay of 253J cells treated with varying concentrations of R11 peptides. (Red: R11; Green: ITGB1; Blue: DAPI). B) Fluorescence assay of T24 cells treated with varying concentrations of R11 peptides. C, D) Western blotting result of Piezo1/integrin β1 signal in 253J and T24 cells treated with varying concentrations of R11 peptides. E) The quantitative result of relative protein expression. F) The result of Ca^2+^ signal and R11 peptides from flow cytometry in T24 cells exposed to R11 peptides. G) The result of Ca^2+^ signal and R11 peptides treated with the agonist (Yoda1 10 nM) and inhibitor (GsMTx4 2.5 nM) of Piezo1. H) Expression levels of Piezo1/integrin β1/YAP signal in T24 cells exposed to Yoda1 or GsMTx4. I) The quantitative analysis of relative protein expression. J, K) Expression levels of proteins in cells knocked down ITGB1 or PIEZO1 and treated with R11 peptides. L, M) The quantitative result of relative protein expression in cells knocked down ITGB1 or PIEZO1.

To elucidate the endocytosis process of R11 peptides, we examined the location of integrin β1 after cells were treated with different peptide concentrations. We observed integrin β1 internalization by both 253J and T24 cell lines in the presence of R11 peptides, and the cellular internalization efficiency increased with higher peptide concentrations (**Figure** **6**A,B). Next, western blotting results showed that R11 peptides activated the Piezo1/integrin β1/YAP signal and RAC protein in a concentration‐dependent manner (Figure 6C,D), with the quantitative data presented in Figure 6E.

The Piezo1/integrin β1 axis mediates the release of Ca^2+^ and subsequent downstream signals.^[^ ^29^, ^30^ ^]^ We then asked R11 peptides influence the Ca^2+^ signal. Flow cytometry results showed that Ca^2+^ signal in T24 cells increased consistently with the concentration of R11 peptides (Figure 6F; Figure S8A, Supporting Information). To further verify the connection between R11 peptides and the Ca^2+^ signal, we applied agonists (Yoda1) and inhibitors (GsMTx4) of Piezo1. Yoda1 enhanced both the uptake efficiency and Ca^2+^ release, while GsMTx4 showed the opposite effect (Figure 6G; Figure S8B, Supporting Information). The western blotting result revealed the expression levels of Piezo1, ITGB1, and YAP in cells following treatment with Yoda1 and GsMTx4 (Figure 6H). Yoda1 treatment led to an enhancement in the expression of proteins, while GsMTx4 treatment resulted in an inhibition of their expression. The quantitative data was depicted in Figure 6I, further confirming the synergistic interplay of Piezo1/ITGB1 axis. These results also indicated that R11 peptides promote Ca^2+^ release through activating Piezo1/ITGB1 signal.

To further explore the correlation between Piezo1/integrin β1 and peptides, T24 and 253J cells with both Piezo1 and integrin β1 knocked down Piezo1 were treated with peptides (Figure 6J,K). The results demonstrated that R11 peptides possessed the capacity to activate the Piezo1/integrin β1 axis and the YAP signaling pathway. It should be noted that the expression of Piezo1/integrin β1/YAP remained synchronous under Piezo1/integrin β1 knockdown conditions. However, R11 could not enhance the expression of Piezo1/integrin β1/YAP when they were knocked down. The quantitative data was presented in Figure 6L,M. These results verified that the Piezo1/integrin β1 axis mediated Ca^2+^ and YAP signal, playing a crucial role in the targeted delivery of R11 peptides.

### 2.7 Integrin β1 Facilitates Delivery in Orthotopic Tumor Models and Clinical Samples

**Figure 7:**
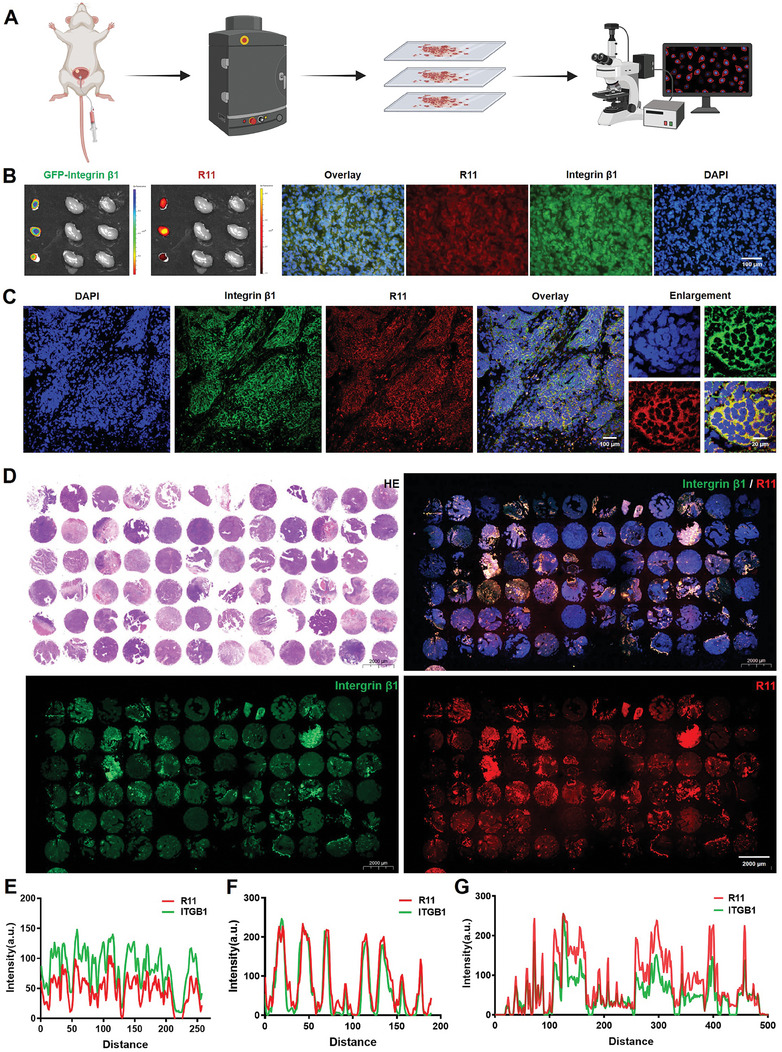
Integrin β1 facilitates delivery in orthotopic tumor models and clinical samples. A) The flow diagram of the animal experiment. B) The IVIS images from mouse orthotopic BC model using integrin β1‐GFP transfected system and images from frozen sections after R11 peptides were injected into the bladder. C) The FISH result in a tissue from an elder patient with T1‐stage BC stained with integrin β1 and R11 peptides respectively. D) The FISH result in a tissue chip from various BC patients stained with integrin β1 and R11 peptides. E) The quantitative data of images in Figure 7B. F) The quantitative data of images in Figure 7C. G) The quantitative data of images in Figure 7D.

Next, we characterized the targeting efficiency of peptides to integrin β1/Piezo1 signal in both in vivo and preclinical experiments. We established a mouse orthotopic BC model using cell lines transfected with integrin β1‐GFP and utilized IVIS system and microscope to visualize the interaction (**Figure** **7**A). R11 peptides were administered via intravesical instillation through the urinary tube, which successfully identified bladder tumors in vivo but not adjacent kidney tissues, as indicated by IVIS results (Figure 7B). Subsequent examination of frozen bladder sections revealed co‐localization between integrin β1‐GFP (Green) and peptides (Red) in the BC orthotopic model. Additionally, a clinical sample from a T1‐stage BC patient demonstrated that R11 peptides effectively bound to integrin β1, accurately identifying tumor tissues (Figure 7C). Additional validation using a BC tissue microarray comprising 72 early‐stage patient samples substantiated the targeting efficiency of R11 peptides, with a consistent expression pattern observed between integrin β1 and R11 peptides across all tissues (Figure 7D). These results underscore the potent binding affinity of R11 peptides for integrin β1, observed in both orthotopic tumor models and clinical tissues, suggesting their potential utility in enhancing tumor‐targeted delivery for cancer therapeutics. The accompanying quantitative data, presented in Figure 7E–G, further reinforce these conclusions.

To further investigate the delivery ability, we then explore whether arginine‐rich peptides could serve as carries for gold nanoparticles, interacting with Piezo1/integrin β1 axis. Gold nanoparticles modified with R11 peptides (Nano‐R11) were assembled, with the particle size increasing proportionally to the concentration of R11 peptides (Figure S9, Supporting Information). Immunofluorescence assays showed that Nano‐R11 increased green fluorescence dots representing endocytic vesicles mediated by integrin β1, including membrane thickening and forming a multilayer structure (Figure S10, Supporting Information). Next, orthotopic models were used to observed that Nano‐R11 identified bladder tumors in vivo, as evidenced by IVIS results (Figure S11A, Supporting Information). Frozen bladder sections still exhibited co‐localization between integrin β1‐GFP and Nano‐R11 (Figure S11B, Supporting Information). These results collectively illustrate that both R11 peptides and R11‐modified nanoparticles are capable of interacting with the Piezo1/integrin β1 axis, demonstrating promising tumor‐targeted imaging and delivery efficiency of peptides as carriers.

## 3 Discussion

**Figure 8:**
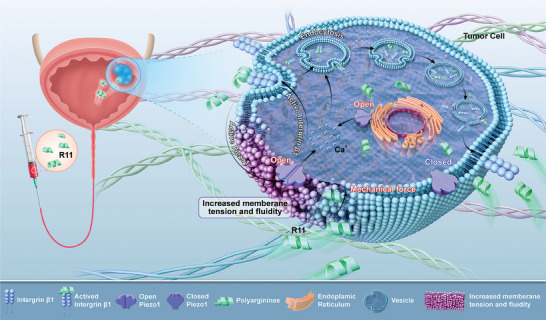
A positive feedback loop orchestrated by mechanical signal axis Piezo1/integrin β1 enhances tumor‐targeted delivery of arginine‐rich peptides.

This study presents a novel approach to enhance tumor‐targeted delivery through the Piezo1/integrin β1 signal axis, involving in the formation and development of membrane fusion. Arginine‐rich peptides were synthesized to facilitate tumor imaging and delivery in BC. The over‐activated Piezo1/integrin β1 signaling axis significantly facilitated tumor‐targeted delivery. R11 peptides directly interacted with integrin β1, promoted integrin recycling and protected integrin β1 from lysosome degradation, establishing a positive feedback loop in delivery. Meanwhile, R11 peptides activated the Piezo1/YAP and Ca^2+^ signal by inducing mechanical fusion and morphological alterations in the membrane, further activating integrin β1 and improving peptides uptake efficiency in cancer cells (**Figure** **8**). The study suggests that targeting the Piezo1/integrin β1 axis initiated a positive feedback loop to enhance tumor‐targeted delivery.

Peptides and cargos typically enter cells through various endocytosis pathways, including macropinocytosis, clathrin‐mediated endocytosis, caveolin‐mediated endocytosis, and clathrin/caveolin‐independent endocytosis.^[^ ^1^ ^]^ It has been demonstrated that peptides may be trapped in the endosomes/lysosomes during the endocytosis process, restricting the targeting effectiveness.^[^ ^5^ ^]^ Our investigations revealed that the macropinocytosis pathway is the preferred route for arginine‐rich peptides. These peptides are efficiently transported into cells via mature vesicles, induced by actin stimulations.^[^ ^31^ ^]^ Initially, peptides interact with membrane proteoglycans to stimulate RAC proteins. This activation triggers F‐actin organization and resulting in actin microfilaments shrinking and the membrane deforming, forming endocytic vesicles that bypass the lysosomal pathway.^[^ ^32^, ^33^ ^]^ Here we found that R11 peptides activated RAC, α‐muscle actin proteins (α‐SMA) microfilaments and F‐Actin, consistent with the macropinocytosis process. In summary, arginine‐rich peptides play a crucial role in enhancing the delivery efficiency to cancer by facilitating the escape of contents from endosomes, a process mediated by Piezo1/integrin β1 axis.

Moreover, our study suggests that arginine‐rich peptides prevent integrin β1 from degradation by lysosome and positively promote its recycling from cytoplasm to membrane according to the fluorescence assay, flow cytometry and western blotting. This recycling process enhances integrin function and delivery within the cell.^[^ ^34^, ^35^, ^36^ ^]^ Integrin β1 recycling is a dynamic and tightly regulated process involving multiple molecular mechanisms, such as the internalization, sorting, and reinsertion of integrin β1 molecules on the cell surface.^[^ ^37^, ^38^ ^]^ The process begins with endocytosis, involving the internalization of the integrin molecule into endosomes on the plasma membrane.^[^ ^39^ ^]^ The internalized integrin β1 is then sorted into early endosomes, where it can either be degraded or recycled back to the plasma membrane.^[^ ^40^ ^]^ The constant flux of integrins between the plasma membrane and cytoplasm allows cells to sense the environment and to adapt their mechanical and morphological properties appropriately. This process is also regulated by various enzymes, such as Rab GTPases and phosphatidylinositol 3‐kinase.^[^ ^41^, ^42^ ^]^ Therefore, investigating novel therapeutic strategies targeting integrin β1 recycling is crucial for tumor delivery.

Arginine‐rich peptides are reported to stimulate dynamic morphological alterations and increase membrane curvature, potentially enhancing the formation and transport of endocytosis vehicles. The insertion of arginine peptides into the lipid bilayer can disrupt its structural integrity, causing local curvature and deformation that may lead to the formation of membrane protrusions or invaginations. The interaction of arginine peptides with the membrane can alter its mechanical properties, such as tension, which is crucial for the activation of mechanosensitive channels like Piezo1.^[^ ^30^, ^43^, ^44^, ^45^ ^]^ Piezo1, as a sensor for membrane tension, regulates downstream Ca^2+^ signals and RAC1 activation during phagocytosis.^[^ ^46^, ^47^ ^]^ Meanwhile, the fusion process induced by nona‐arginine(R9) exhibited regions of protrusions, bifurcations, and multilamellarity, suggesting a strong curvature‐generating interaction, analogous to the vesicle fusion induced by Ca^2+^.^[^ ^19^ ^]^ Similarly, dodeca‐arginine (R12) peptides induced dynamic deformation and topical inversion of the plasma membrane, suggesting the involvement of membrane fusion or hemifusion.^[^ ^20^ ^]^ Membrane deformation induced by arginine peptides could trigger signaling cascades that lead to cytoskeletal rearrangements, resulting in morphological alterations. Here, we confirmed that R11 peptides induce multilayered structures and protrusions in the membrane after interacting with the integrin β1, further activating the Piezo1/YAP and Ca^2+^ signal, ultimately promoting mechanical delivery to cancer and offering possibilities for drug synthesis and modification in cancer therapy.

This study unveils a novel mechanism of the integrin β1/Piezo1 activation/Ca^2+^ influx and YAP pathway in peptide delivery. The positive feedback regulation loop, encompassing membrane morphological alterations, Piezo1/integrin β1 axis, and YAP/Ca^2+^ signal, reinforces tumor‐targeted delivery. Xu et al. also reported a mechanical positive feedback loop that the engagement of integrin β1 could induce local changes in membrane tension, which in turn activated Piezo1^[^ ^23^ ^]^. Piezo1 opened in response to membrane deformation, allowing the influx of Ca^2+^, which acted as a second messenger and modulated the activity of YAP. YAP has been shown to modulate the expression of genes that affect integrin function and ECM remodeling, which reinforced the initial activation of integrin β1, creating a positive feedback loop. Therefore, targeting Piezo1/integrin β1/YAP axis may be a promising strategy for cancer treatment. Preclinical studies have demonstrated that Piezo1, integrin, and YAP inhibitors significantly suppress tumor growth and metastasis, and some of the small molecule PROTACs have been evaluated in clinical trials.^[^ ^48^, ^49^, ^50^ ^]^ However, we acknowledged the limitations of our current findings and the need for additional research to fully elucidate the complex interactions between Piezo1, integrin β1, and YAP signaling. We anticipate the development of promising drugs or peptides for cancer therapy targeting the Piezo1/integrin β1 axis.

## 4 Conclusion

Arginine‐rich peptides play a pivotal role in enhancing mechanical sensing and cellular uptake through the Piezo1/integrin β1 axis, thereby establishing a positive feedback loop that ultimately promotes tumor‐targeted delivery. The over‐activated Piezo1/integrin β1 signaling axis significantly facilitates tumor‐targeted delivery. Furthermore, R11 peptides formed hydrogen bonds with integrin β1, which not only promotes integrin recycling from cytoplasm to membrane but also prevents the degradation. Additionally, R11 peptides induce the formation of multilayer structures and sprouting protuberances in the membrane, and further enhance the delivery by activating Piezo1//YAP/Ca^2+^ signal. The Piezo1/integrin β1 signaling axis contributes significantly to the targeting efficiency of R11 peptides in both orthotopic tumor models and clinical samples, demonstrating satisfactory tumor‐targeted imaging efficiency both in vivo and in vitro. In summary, the Piezo1/integrin β1 signaling axis orchestrates a positive feedback loop that strengthens the tumor‐targeted delivery of arginine‐rich peptides and offering novel insights for cancer diagnosis and treatment.

## 5 Experimental Section

### Materials and Regents

R11 peptides (GRRRRRRRRRRR‐TRITC) were synthesized and purified by GL Biochem Ltd (Shanghai, China). The sequences of oligonucleotides and primers were synthesized by Sangon Biotech (Shanghai) Co., Ltd. and were shown in Table S1 (Supporting Information). The PrimerScript RT reagent kit and SYBR Green Master Mix were purchased from TAKARA Biotechnology Co. (Dalian, China). Chlorpromazine (CPZ), Methyl‐β‐cyclodextrin (MβCD), Dynasore, Cycloheximide (CHX), MG132, Primaquine, Chloroquine (CQ) and CCK‐8 reagent were purchased from TargetMol (USA). The agonists (Yoda1) and inhibitors (GsMTx4) of PIEZO1 and EIPA were purchased from MedChemExpress (China). The integrin β1 plasmid and PIEZO1 overexpressed plasmid was synthesized by Biokeeper (China). The cell culture medium was purchased from Procell Life Science & Technology Co., Ltd. All the chemicals were of analytical grade and were used without further purification.

### Cell Culture

The human bladder cancer cell lines 5637, J82, T24 and 253J were purchased from the American Type Culture Collection (Manassas, VA, USA), and the normal control cell SVHUC which was the SV‐40 immortalized human uroepithelial cell line was obtained from the American Type Culture Collection (ATCC, USA). J82 and 5637 cells were cultured in RPMI 1640 medium supplemented with 10% FBS under a humidified 5% CO_2_ and 95% air atmosphere at 37 °C. SV‐HUC, T24 and 253J cells were cultured in DMEM medium supplemented with 10% FBS under a humidified 5% CO_2_ and 95% air atmosphere at 37 °C.

### Fluorescence In Situ Hybridization

All clinical tissues were obtained and carried out following guidelines approved by the ethics committee of first affiliated hospital of Xi'an Jiaotong University, Xi'an, China. The informed written consent from all participants was obtained prior to the research. Tissue sections were prepared on positively charged slides and subjected to an overnight heating process. Following this, they were deparaffinized in xylene and sequentially dehydrated through a gradient of absolute ethanol concentrations, concluding with air‐drying. The sections were then briefly boiled in a repair solution for 10–15 minutes, after which they were allowed to cool naturally. Subsequently, a working solution of proteinase K at a concentration of 20 µg mL^−1^ was applied to the slides, and they were incubated at 37 °C for 15 minutes. Next, 3% methanol‐H_2_O_2_ was added, and the slides were incubated in the dark at room temperature for 15 min. The slides were washed with PBS (pH 7.4) two times for 3 min per wash, with gentle agitation and protected from light. A prehybridization solution was added to each section and incubated for 1 h at 37 °C. The R11 probe hybridization solutions (3 µg mL^−1^) or integrin β1 antibody were combined overnight in a thermostat. The hybridization solution was removed and sections were washed with 2 × SSC for 5 min, 1 × SSC two times for 5 min, and 0.5 × SSC for 10 min at room temperature. Blocking serum containing BSA was added to the sections and incubated at room temperature for 30 min. The sections were dried slightly, freshly prepared DAPI chromogenic reagent was added to the stained tissue, and the sections were mounted with resin mounting medium. The results were imaged using a fluorescence microscope.

### Cellular Uptake Pathways

Flow cytometry was used to measure the fluorescence signal. Cells were seeded in a 6‐well plate at 1.0 × 10^5^ cells per well and treated with R11 peptides and a series of inhibitors for 4 h, including NaN3 (active uptake inhibitor, 20 mM), methyl‐β‐cyclodextrin (MβCD: lipid raft‐mediated endocytosis inhibitor, 5 µg mL^−1^), EIPA (macropinocytosis inhibitor, 10 µmol mL^−1^), Chlorpromazine (CPZ: clathrin‐mediated endocytosis, 5 µg mL^−1^) or Dynasore (dynamin‐dependent endocytosis inhibitor, 80 µmol mL^−1^) in serum‐free media at 37 °C or 4 °C for 4 h, respectively. Then, the cells were trypsinized, washed, and suspended in 1 mL PBS. The fluorescence intensity of 10 000 cells was measured using a BD LSR II flow cytometer.

### Real‐Time Quantitative PCR

Real‐time quantitative PCR was performed to evaluate the expression of ITGA2, ITGB1, and PIEZO1 mRNA expression. SVHUC‐1, 5637, J82, T24, and 253J cells were seeded into 6‐well plates and cultured in a growth medium overnight. The total RNA was extracted using Fast200 reagent following the manufacturer's protocol. Subsequently, cDNA was synthesized using a PrimerScipt RT reagent kit (Takara Bio). In addition, the relative levels of the target gene mRNA transcript were measured with SYBRGreen PCR Master Mix (Takara Bio, Dalian, China) using RT‐PCR (C1000 Thermal Cycler; Bio‐Rad Laboratories Inc., Hercules, USA). All of the samples were run in triplicate and normalized to 18S. The sequence of primers was exhibited in Table S1 (Supporting Information).

### Western Blotting Assay

The total proteins were isolated with RIPA lysis buffer (Beyotime, China) with a protease inhibitor, phosphatase inhibitor, and 0.1 M PMSF (Beyotime, China). Membrane and cytoplasmic proteins were extracted using a Nuclear Extraction Kit (Beyotime, P0033, China). The extract of proteins and western blotting assays were performed as usual. Anti‐integrin α2 (A19068), PIEZO1 (A4340), YAP (A1002), and β‐Actin (AC026) were purchased from Abclonal. Anti‐integrin β1 (ab52971), integrin αV (ab179475), integrin β3 (ab119992), p‐FAK (ab81293), p‐SRC (ab185617), RAC (ab155938) were purchased from ABCAM.

### Transfection and Lentiviral Infection

The small interfering RNA(si‐RNA) knocked down integrin β1 and PIEZO1 was purchased from Genepharma Technology (China). GP‐Transfect‐Mate was used to transfect siRNA into T24 and 253J cells. Lentivirus‐overexpressed integrin β1, PIEZO1, and empty lentivirus vectors were obtained from Biokeeper (China) and GeneChem Company. The lentiviruses were packaged to improve the transfection efficiency according to the procedure. The cells were screened using puromycin and used for subsequent experiments after lentivirus transfection for 48 h. In the CHX assay, cells were treated with CHX (100 µM) and R11 peptides for 0, 4, 8, 12, 24 h. Cells were treated with MG132 (10 µM) and CQ (20 µM) for 24 h.

### Molecular Docking

The PDB format of integrin β1 and PIEZO1 were obtained from https://www.rcsb.org. Chimera was used to build 3D models of R11 peptides and integrin β1, and Autodock software was used for protein‐peptide docking. In the pre‐docking process, polar ammonia atoms were added first, and then charge correction was performed by calculating Gasteiger. Then, the parameters of the docking box were adjusted to wrap the protein completely at the center, and the docking was performed by the Lamarckian Genetic Algorithm. Other parameters used the default values.

### ELISA Assay

ELISA assay was conducted to ascertain the concentration of R11 peptides necessary for binding to integrin β1 and eliciting half of the maximum fluorescence intensity response. The specific integrin β1 ELISA kit, utilized for this purpose, was procured from Shanghai Westang Bio‐Tech CO., LTD. The assay procedure commenced with the addition of integrin β1 standards to the microplate wells, prepared at a concentration of 0.5 µg mL^−1^, in triplicate. Following this, R11 peptides were serially diluted in PBS to final concentrations of 0.001, 0.01, 0.1, 1, 10, 100, 1000, and 10 000 nM. These dilutions were then separately introduced into the microplate wells and incubated for a duration of 2 hours at a temperature of 37 °C. Subsequently, any unbound R11 peptides were eliminated through a rigorous washing process using washing buffer to ensure complete removal of non‐specifically bound peptides. The microplate was then analyzed using a microplate reader, which quantified the intensity of the fluorescence signal emitted by the bound peptides and allowed for the determination of the concentration‐response curve. Lastly, half‐maximal effective concentration (EC50) could be calculated.

### Immunofluorescence

Cells were planted in the glass coverslips coated with fibronectin and treated with R11 peptides at different times, then fixed with 4% paraformaldehyde (PFA) in PBS for 15 minutes at room temperature. Add the integrin β1 antibody (P5D2, ABCAM, ab24693), PIEZO1 antibody (Abclonal, A4340), YAP antibody (Abclonal, A1002), α‐SMA (Abclonal, A7248), or Phalloidin (Abclonal, RM02836) and place them in the humidifying box at 4 °C overnight (1:200). After washing with PBS, homologous secondary antibodies were added to the coverslips at room temperature for 1 h. DAPI was added to stain the nucleus at room temperature for 5 min. After washing, the slices were sealed with an anti‐fluorescence quenching reagent and observed using fluorescence microscope.

### Antibody Block Assay

Antibody integrin α2β1 (ab30483) used for blocking the binding motif was purchased from ABCAM. Cells were planted in the 6‐well plates and treated with Anti‐integrin α2β1 (5 ug/mL). After incubation for 2 h, the cells were treated with R11 peptides and collected. Then, the cells were trypsinized, washed, and suspended in 1 mL PBS. The fluorescence intensity of 10 000 cells was measured using flow cytometer.

### Cell Surface Integrin β1 Labeling and Recycling Assay

For the experimental treatment with primaquine, the compound was diluted in PBS to a final concentration of 100 µM and incorporated into the pre‐warmed culture medium. Cells were pre‐treated in serum‐free medium, on ice for 30 minutes. Cells were treated with either the 12G10 antibody, which targets active integrin β1 (sourced from ABCAM, ab30394); or the MAB13 antibody, specific for inactive integrin β1 (provided by BD Biosciences, 552 828). Both antibodies were diluted at 1:500 in serum‐free medium on ice for 30 minutes. This step allows for the specific labeling of both active and inactive pools of integrin β1 present on the cell surface. Following incubation, any unbound antibody was removed by washing the cells with cold PBS, effectively initiating the endocytosis process. To trigger endocytosis, pre‐warmed serum‐free medium containing R11 peptides was added to the cells, and the cells were incubated at 37 °C for periods of 15 and 30 minutes. For the 0‐minute control group, cells were maintained on ice and cold medium was added to prevent endocytosis. After the incubation, the warm medium was removed, and the cells were washed again with cold PBS. Cells were then detached by gently scraping and fixed with 4% paraformaldehyde (PFA) in PBS for 15 minutes at room temperature. Following fixation, cells were washed with PBS and incubated with the appropriate secondary antibody, diluted 1:200 in PBS, for 1 hour at 4 °C. Finally, cells were washed again with PBS and prepared for analysis via flow cytometry and confocal microscopy.

### Cell Membrane and Cytoplasmic Protein Extraction

Cell membrane protein and cytoplasmic protein extraction kit was purchased from Beyotime. Cells were collected and mixed with membrane protein extraction reagent A containing PMSF, set at 4 °C for 15 minutes, and then broken by ultrasound. Centrifuge at 700 g at 4 °C for 10 minutes and collect the supernatant into the centrifuge tubes. Centrifuge again at 4 °C, 14 000 g for 30 minutes, and collect the supernatant to get cytoplasmic protein. Then centrifuge at 14 000 g for 10 seconds and discard the supernatant. Add 200 µl of membrane protein extraction reagent B, suspend precipitation through violent shock and ice bath for 10 minutes. Subsequently, centrifuge at 14 000 g for 5 minutes and collect the supernatant, which was the membrane protein solution.

### Transmission Electron Microscope (TEM)

T24 cells were incubated with R11 peptides for 1 h to prepare microtome samples of cells. After that, cells were carefully washed with sterile PBS twice and transferred into 1.5 mL eppendorf tubes. Collected cell pellets were resuspended in a solution of 4 vol % paraformaldehyde and 1.25 vol % glutaraldehyde and were fixed overnight. Then, samples were postfixed in a 2 vol % osmium tetroxide solution for 45 min. After this, cells were fully dehydrated and embedded in epoxy resin. Ultrathin sections of 70 nm were cut and poststained with uranyl acetate and lead citrate. Cell samples were analyzed by TEM at 100 kV.

### In Vivo experiments

All the animal experiments were carried out following guidelines approved by the ethics committee of Xi'an Jiaotong University, Xi'an, China. Female nude mice aged 4–6 weeks were randomly sorted. After gaseous anesthesia, an intravenous indwelling needle lubricated with paraffin cotton was inserted into the bladder and fixed well. Then, the urine was removed, and the bladder was rinsed with PBS 3 times. Next, the abdominal skin was disinfected by iodine tincture and cut open for approximately 1 cm. GFP‐integrin β1 cells (1 × 10^6^/mL) were injected into the bladder wall with an insulin needle under direct visualization. Last, the abdominal wall was stitched and bound with gauze. After 7 days, the effect of bladder tumor implantation was observed. After intravesical perfusion of R11 peptides or gold nanoparticles, tumor‐bearing mice were detected using an in vivo imaging system/IVIS (AniView) to take images. In addition, frozen sections of the tumors were stained with DAPI, and the fluorescence of each section of the tumor was analyzed by fluorescence microscopy.

### Statistical Analysis

Data were expressed as the means ± standard deviation (SD). Significant differences were determined using Student's t‐test or one‐way ANOVA as appropriate. A 2‐tailed *p* < 0.05 was considered statistically significant. *p < 0.05; **p < 0.01; ***p < 0.001. All data were analyzed with GraphPad Prism software.

## Conflict of Interest

The authors declare no conflict of interest.

## Supporting information



Supporting Information

## Data Availability

The data that support the findings of this study are available from the corresponding author upon reasonable request.
