# Supplementary material for: Enhanced Tumor‐Targeted Delivery of Arginine‐Rich Peptides via a Positive Feedback Loop Orchestrated by Piezo1/integrin β1 Signaling Axis
Source: Adv Sci (Weinh). 2024 Sep 11;11(42):2409081. doi: 10.1002/advs.202409081 (PMC11558097; doi:10.1002/advs.202409081)
Supplement: Supplementary file 1 — Supporting Information [file ADVS-11-2409081-s001.pdf]

ADVANCED SCIENCE
Open Access

Supporting Information

for *Adv. Sci.*, DOI 10.1002/advs.202409081

Enhanced Tumor-Targeted Delivery of Arginine-Rich Peptides via a Positive Feedback Loop Orchestrated by Piezo1/integrin β1 Signaling Axis

Minghai Ma, Xing Li, Minxuan Jing, Pu Zhang, Mengzhao Zhang, Lu Wang, Xiao Liang, Yunzhong Jiang, Jianpeng Li, Jiale He, Xinyang Wang, Min Lin, Lei Wang* and Jinhai Fan**

Enhanced tumor-targeted delivery of arginine-rich peptides via a positive feedback loop orchestrates by Piezo1/ integrin β1 signaling axis

Minghai Ma, Xing Li, Minxuan Jing, Pu Zhang, Mengzhao Zhang, Lu Wang, Xiao Liang, Yunzhong Jiang, Jianpeng Li, Jiale He, Xinyang Wang, Min Lin*, Lei Wang*, and Jinhai Fan*

M. Ma, M. Jing, P. Zhang, M. Zhang, L. Wang, Y. Jiang, J. Li, J. He, X. Wang, J. Fan
Key Laboratory of Environment and Genes Related to Diseases, Ministry of Education
Department of Urology
The First Affiliated Hospital
Xi'an Jiaotong University
Xi'an 710061, China
Email: fanjinhai@xjtu.edu.cn

X. Li, X. Liang, L. Wang
Department of Thoracic Surgery
Tangdu Hospital
Air Forth Medical University
Xi'an 710038, China
Email: tuodi86@fmmu.edu.cn

M. Lin
Key Laboratory of Biomedical Information Engineering, Ministry of Education
Bioinspired Engineering and Biomechanics Center (BEBC)
School of Life Science and Technology
Xi'an Jiaotong University
Xi'an 710049, China
Email: minlin@xjtu.edu.cn

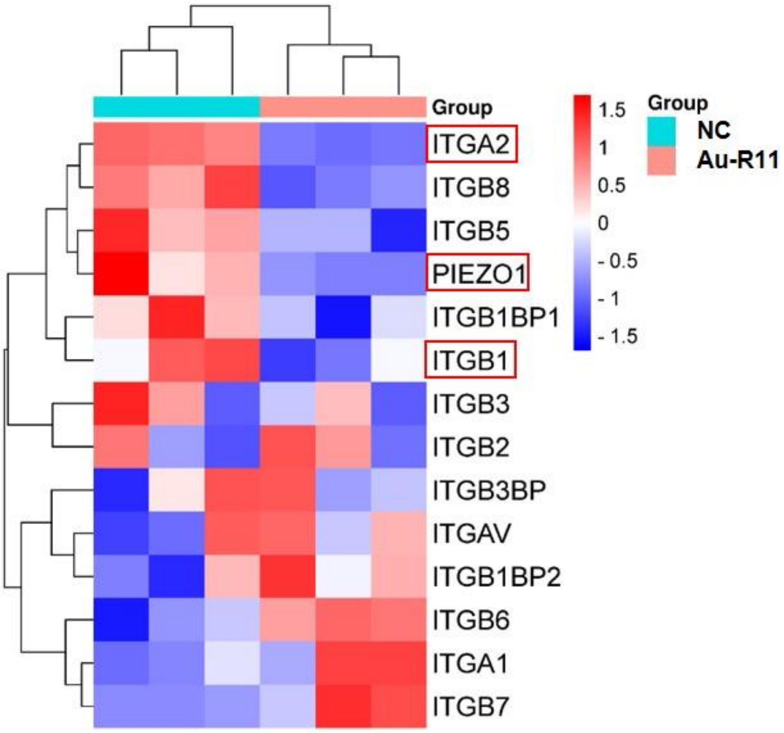

Figure S1. RNA-sequencing results of cells treated with R11 peptide-assembled nanoparticles (Au-R11).

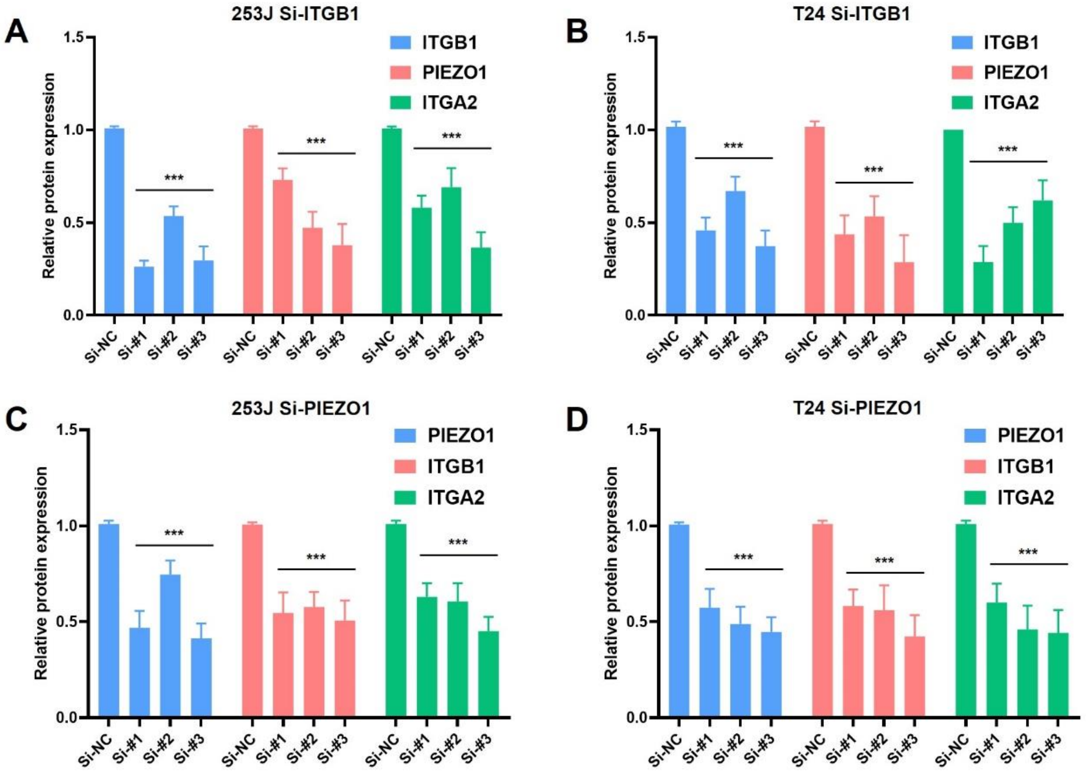

Figure S2. The quantitative data of expression level of ITGB1, PIEZO1, ITGA2 after knocking down ITGB1 or PIEZO1 in 253J and T24 cell lines.

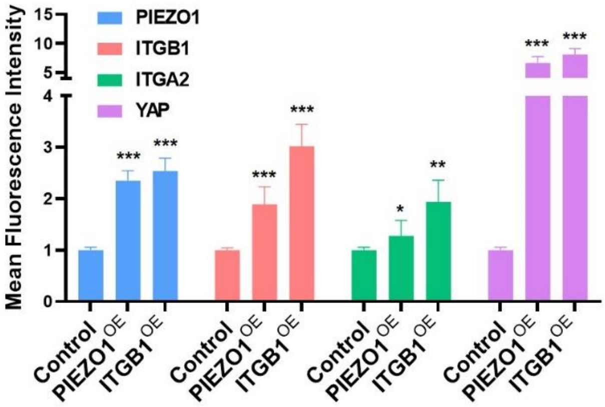

Figure S3. The quantitative of western blotting result in SV-HUC cells overexpressed PIEZO1 and ITGB1.

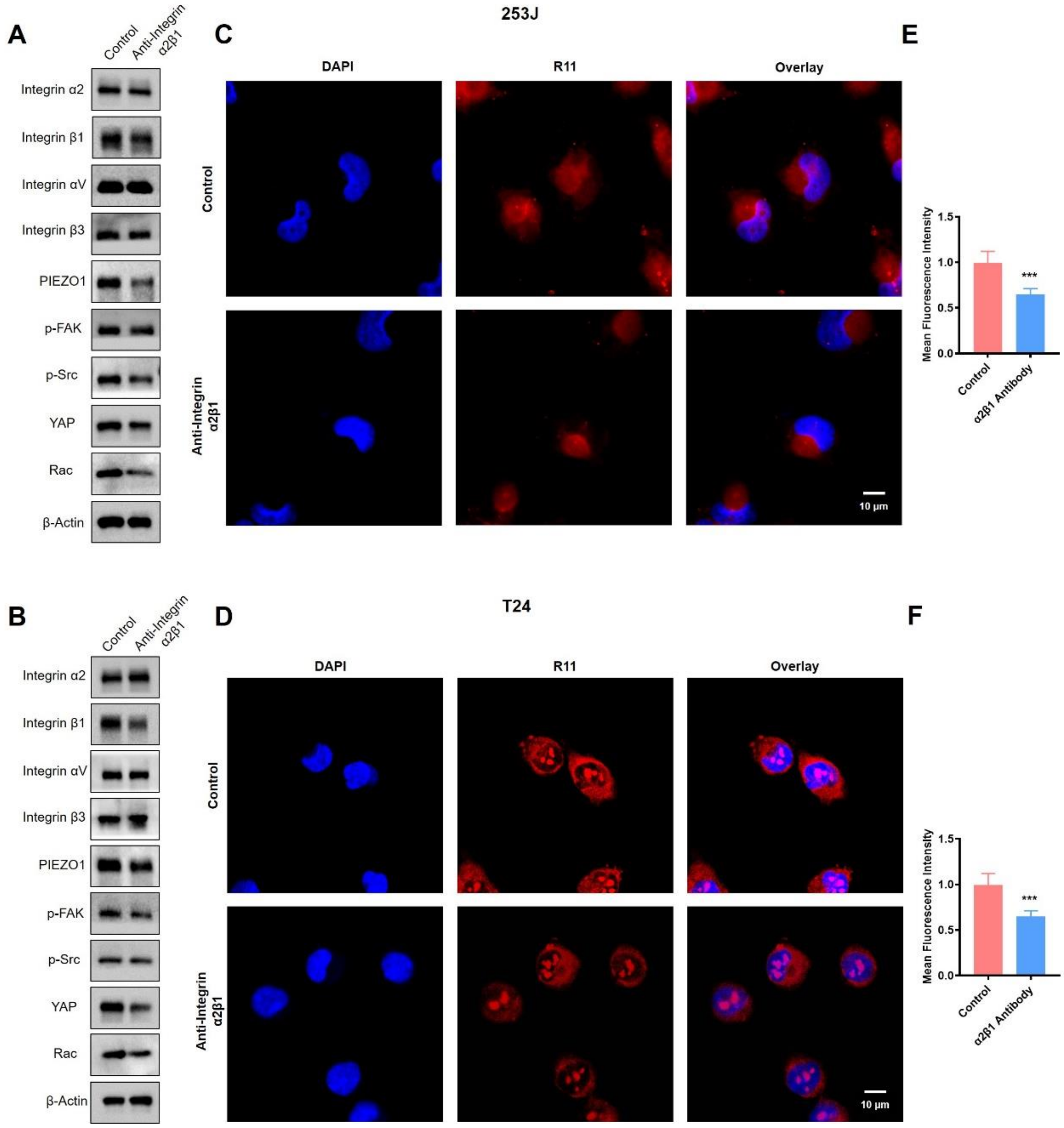

Figure S4. A-B) Expression level of integrins, PIEZO1, p-FAK, p-Src, YAP, Rac from western blotting in 253J and T24 cells after blocking integrin α2β1. C-D) The uptake ability of peptides from fluorescence assay when integrin α2β1 was blocked in 253J and T24 cells. E-F) The quantitative data of fluorescence assay in 253J and T24 cells.

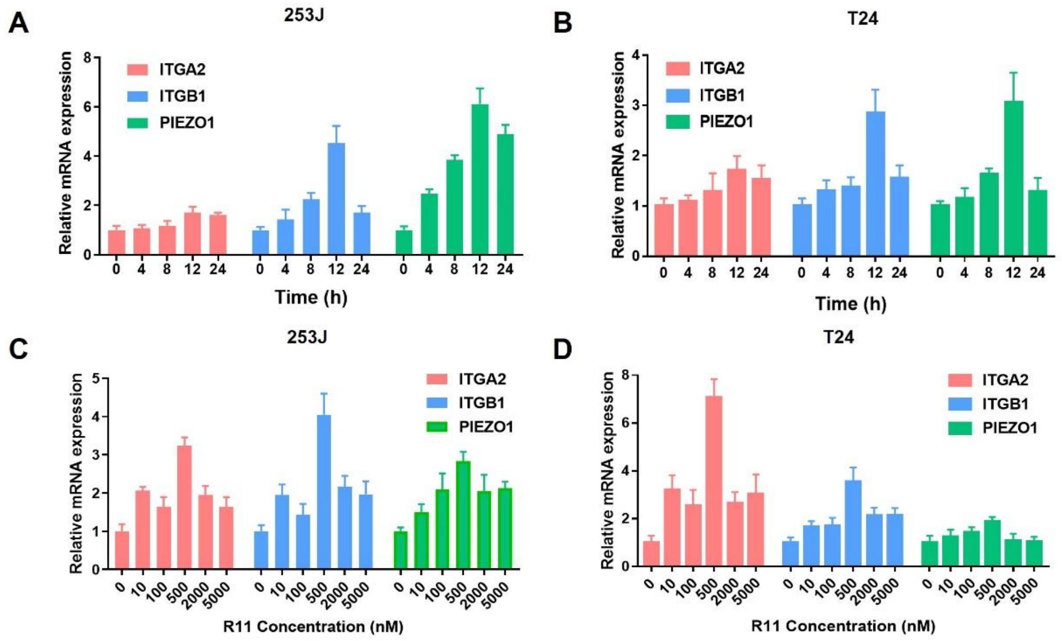

Figure S5. PCR analysis of ITGA2, ITGB1 and PIEZO1 of 253J and T24 cells in a time-dependent and concentration-dependent manner.

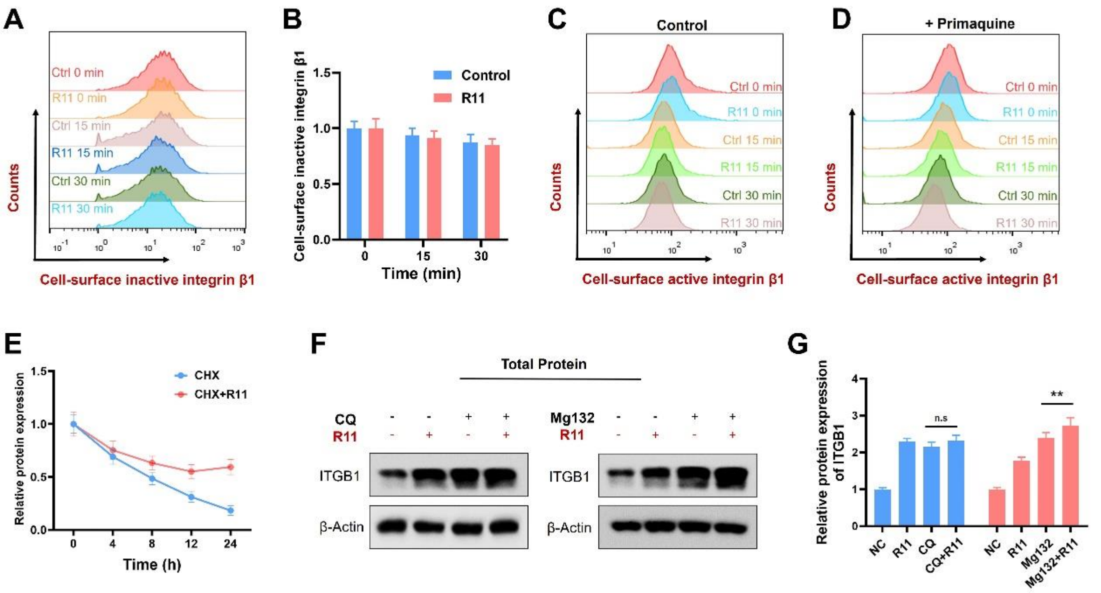

Figure S6. A) The cell-surface levels of active integrin β1 from flow cytometry following the initiation of endocytosis at 15 and 30 minutes, with or without R11 peptides present. B) The cell-surface levels of active integrin β1 from flow cytometry following the initiation of endocytosis at 15 and 30 minutes, with the presence of primaquine. C) The cell-surface levels of inactive integrin β1 from flow cytometry after endocytosis was triggered. D) The quantitative result of the cell-surface levels of inactive integrin β1. E) The quantitative data of degradation cycle in cells treated with CHX and R11 peptides. F) The expression level of integrin β1 with the presence of CQ or Mg132 and R11 peptides for 24 h. G) The quantitative data of western blotting result.

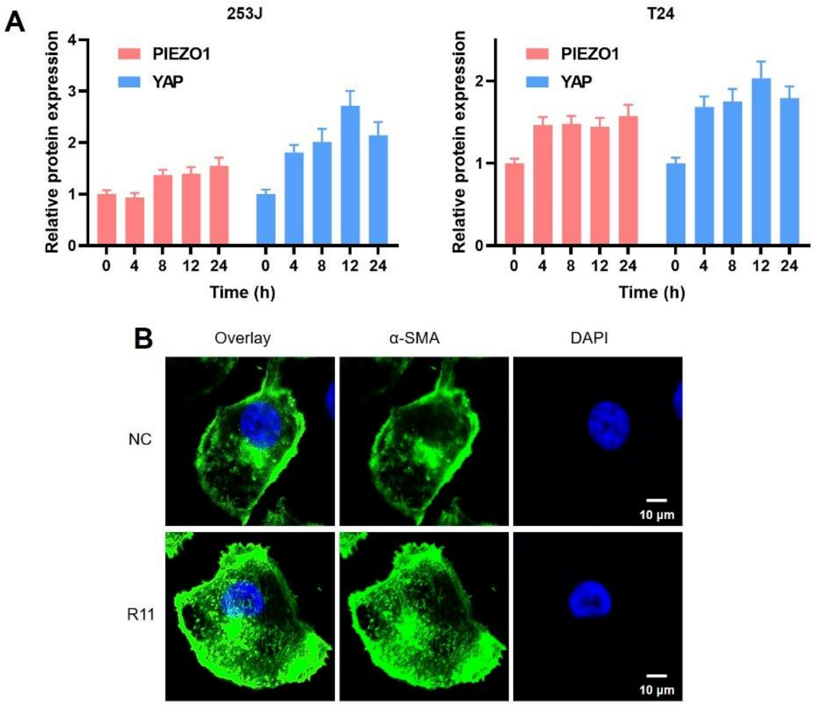

Figure S7. A) The relative protein expression level of PIEZO1 and YAP in 253J and T24 cells treated with R11 peptides. B) The fluorescence of staining for α-SMA in T24 cells treated with R11 peptides.

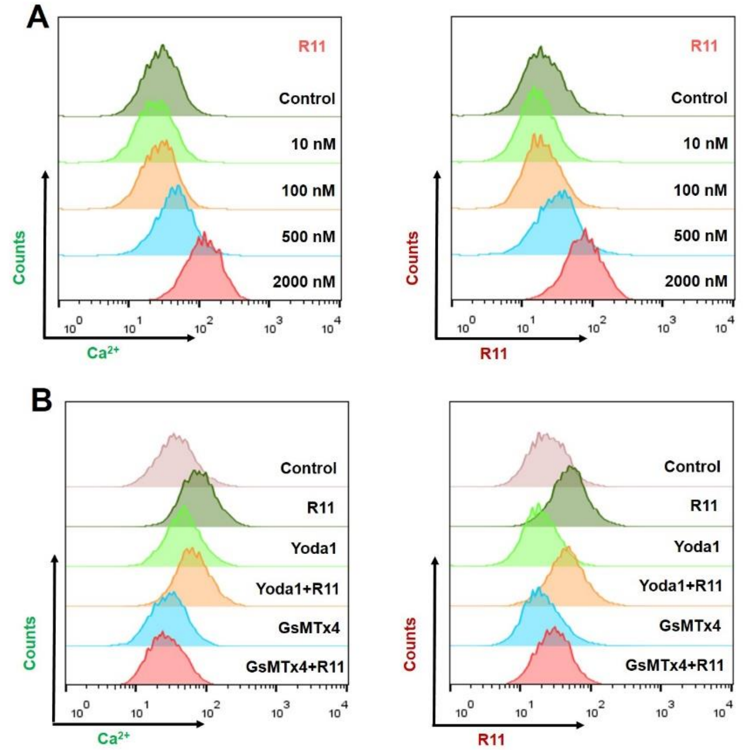

Figure S8. A) The Ca^{2+} signal and peptides signal from flow cytometry in T24 cells with the treatment of R11 peptides. B) The Ca2+ signal and peptides signal from flow cytometry in T24 cells with the treatment of Yoda1 and GsMTx4.

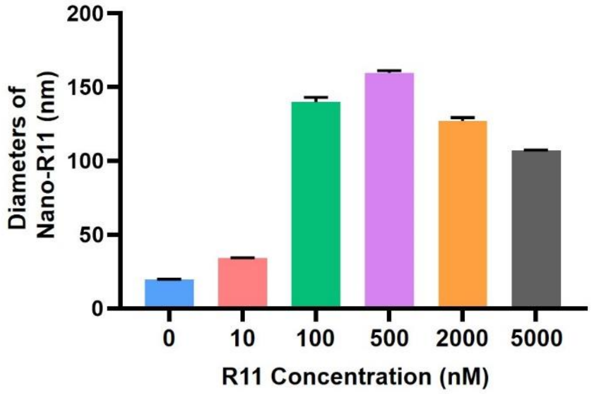

Figure S9. The diameter of gold nanoparticles modified with R11 peptides (Nano-R11) with different concentrations (0, 10, 100, 500, 2000, 5000 nM).

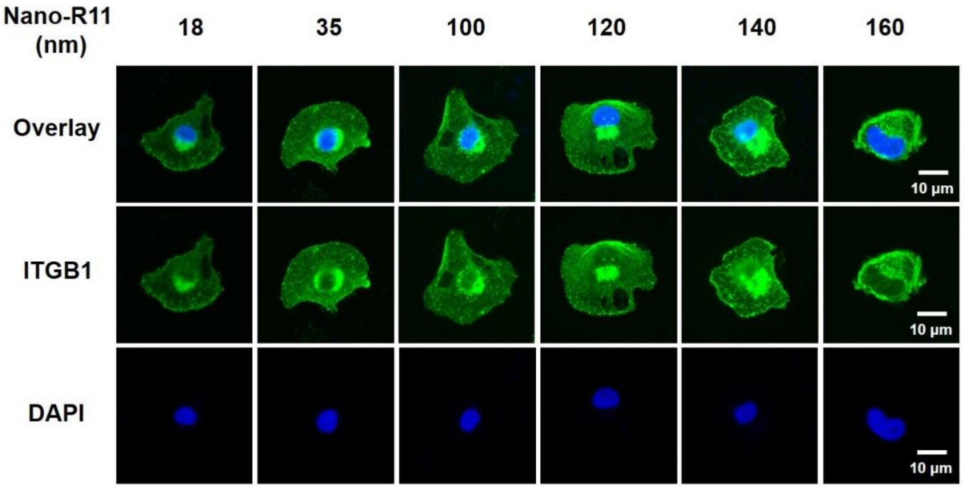

Figure S10. Fluorescence assay of cells treated with peptide-assembled nanoparticles (Nano-R11) with different particle sizes.

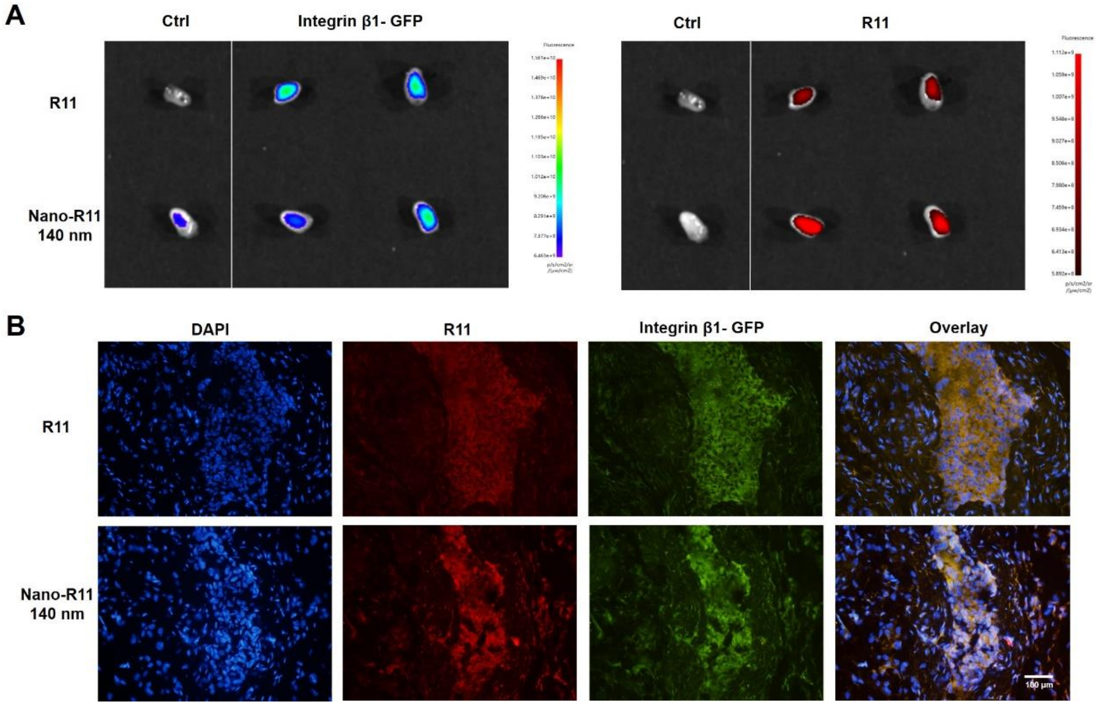

Figure S11. Piezo1/integrin β1 enhanced the delivery of peptide-assembled nanoparticles. A) The IVIS images from mouse orthotopic BC model using Integrin β1-GFP transfected system after R11 peptides and Nano-R11 (140 nm) were injected into the bladder through intravesical instillation. B) The frozen sections from mouse orthotopic BC model using Integrin β1-GFP transfected system after R11 peptides and Nano-R11 (140 nm) were injected into the bladder through intravesical instillation. (Red: R11; Green: Integrin β1; Blue: DAPI)

Table S1. The sequence of gene-specific primers

ITGB1 Reverse	CCTTTGCTACGGTTGGTTACATT
ITGB1 Forward	CCTACTTCTGCACGATGTGATG
ITGA2 Reverse	AGTAACCAGTTGCCTTTTGGATT
ITGA2 Forward	CCTACAATGTTGGTCTCCCAGA
PIEZO1 Reverse	GGGCACAATATGCAGGCAGA
PIEZO1 Forward	GGACTCTCGCTGGTCTACCT
18S Reverse	TAGTAGCGACGGGCGGTGTG
18S Forward	CAGCCACCCGAGATTGAGCA
